# Neuroinflammation in Schizophrenia: The Key Role of the WNT/β-Catenin Pathway

**DOI:** 10.3390/ijms23052810

**Published:** 2022-03-04

**Authors:** Alexandre Vallée

**Affiliations:** Department of Clinical Research and Innovation (DRCI), Foch Hospital, 92150 Suresnes, France; alexandre.g.vallee@gmail.com

**Keywords:** neuroinflammation, oxidative stress, schizophrenia, WNT/β-catenin pathway, glutamate, PPARγ, PPARα

## Abstract

Schizophrenia is a very complex syndrome involving widespread brain multi-dysconnectivity. Schizophrenia is marked by cognitive, behavioral, and emotional dysregulations. Recent studies suggest that inflammation in the central nervous system (CNS) and immune dysfunction could have a role in the pathogenesis of schizophrenia. This hypothesis is supported by immunogenetic evidence, and a higher incidence rate of autoimmune diseases in patients with schizophrenia. The dysregulation of the WNT/β-catenin pathway is associated with the involvement of neuroinflammation in schizophrenia. Several studies have shown that there is a vicious and positive interplay operating between neuroinflammation and oxidative stress. This interplay is modulated by WNT/β-catenin, which interacts with the NF-kB pathway; inflammatory factors (including IL-6, IL-8, TNF-α); factors of oxidative stress such as glutamate; and dopamine. Neuroinflammation is associated with increased levels of PPARγ. In schizophrenia, the expression of PPAR-γ is increased, whereas the WNT/β-catenin pathway and PPARα are downregulated. This suggests that a metabolic-inflammatory imbalance occurs in this disorder. Thus, this research’s triptych could be a novel therapeutic approach to counteract both neuroinflammation and oxidative stress in schizophrenia.

## 1. Introduction

Schizophrenia is a neurodevelopmental and mental illness affecting nearly 1% of the world’s population, and the age of onset age is between 23 and 34 years in women and close to 30 years in men [1]. Schizophrenia is characterized by generalized brain multi-dysconnectivity and marked by abnormal brain development, deregulated neuronal migration, impaired spatial neural arrangement, and the absence of gliosis [2]. This mental disorder is composed of cognitive, behavioral, and emotional disorders, and to be diagnosed with schizophrenia, patients should have two (or more) of the following negative symptoms for a minimum of six months—disorganized speech, grossly disorganized or catatonic behavior—and at least one positive symptom [3]. Positive symptoms are composed of delusions and hallucinations, while negative symptoms are deficits in normal behavior, such as alogia, associability, blunted affect, or anhedonia [4]. The pathophysiological process of the onset of schizophrenia remains unknown to this day. Many signaling pathways have been researched in attempts to explain the neurodevelopmental pathology of this disorder, with genetic, neurodevelopmental, and neurochemical hypotheses being proposed [5]. One possible hypothesis postulates that schizophrenia may be caused by changes (i.e., neuroinflammation and oxidative stress) in the fetus. However, this hypothesis remains both uncertain and unclear.

Recent investigations have observed that the inflammation of the central nervous system (CNS) and immune dysfunction may have a role in the pathogenesis of schizophrenia. This hypothesis developed from immunogenetic evidence and the high propensity of schizophrenic patients to develop autoimmune diseases [6,7]. Neuroinflammation can cause white matter damage and dysconnectivity, therefore generating the appearance of symptoms related to schizophrenia [6].

The WNT/β-catenin pathway is mainly involved in the control of cell development and regeneration, as well as tissue homeostasis [8,9]. This pathway is also associated with several other psychiatric diseases and disorders [10,11,12,13]. The disruption of the WNT/β-catenin pathway is associated with the occurrence of chronic neuroinflammation in several diseases, in particular schizophrenia [11,14]. 

Understanding the complexity of the relationship between the WNT/β-catenin pathway and neuroinflammation in schizophrenia will help to identify new therapeutic targets. Thus, this review focuses on describing the main role of neuroinflammation in schizophrenia and its relationship with the WNT/β-catenin pathway, which leads to the development of oxidative stress, and highlighting possible therapeutic targets. 

## 2. Neuroinflammation in Schizophrenia

The interrelationship between the immune and neuroinflammatory systems plays a major role in the neurobiology of schizophrenia [15]. Recent research has shown that chronic neuroinflammation affecting the CNS could be associated with schizophrenia [16]. Indeed, a significant association was observed between schizophrenia and the expression of the major histocompatibility complex (MHC), located on chromosome 6, in brain and immune cells involved in adaptive immunity (CD19 and CD20B lymphocytes) [17]. Moreover, the overstimulation of dopamine D3 receptors and interferon γ synthesis (IFNγ) in lymphocytes has been observed in unmedicated schizophrenic patients [18]. 

Several studies, including meta-analyses, have found immune dysfunction in schizophrenia [19,20]. In contrast, other studies have observed immunological modification in only 40% of schizophrenic patients [21,22]. It remains important to define efficient immune biomarkers to identify schizophrenic patients who could benefit from anti-inflammatory treatment [23]. In schizophrenic disorder, several studies have shown that the secretion of pro-inflammatory and anti-inflammatory cytokines is concomitantly disrupted [24]. Cytokines represent a wide range of molecules generated by cells, such as B and T immune cells, lymphocytes, macrophages, endothelial cells, and fibroblasts. In the first psychotic episode of schizophrenia, a decrease in anti-inflammatory markers, such as interleukins IL-10 and IL-4, has been observed, while pro-inflammatory factors, such as IL-6, are increased [1]. The oversecretion of cytokines has also been observed in peripheral blood mononuclear cells, with the stimulation of the expression of IL-6, IL-8, and tumor necrosis factor α (TNF-α) and a decreased expression of IL-2mRNA [21]. In the cerebrospinal fluid (CSF) of schizophrenic patients, the dysregulation in the expression of cytokines has also been observed [25], with increased levels of IL-1, IL-6, and IL-8 [17]. In addition, a relationship has been observed between the levels of IL-6, TNF-α, and IFNγ; the high risk of subclinical psychotic symptoms with a marked decrease in their expression under antipsychotic treatment has also been seen [17]. 

Many inflammatory endothelial cell molecules are involved in schizophrenic disorders, including creatine kinase m/B, angiotensin-converting enzyme, matrix metalloproteinase, thyroid-stimulating hormone, thyroxine-binding globulin, intercellular adhesion molecule 1, cortisol, macroglobulin α-2, and thrombopoietin. The altered expression of these molecules contributes to the activation of the acute phase response, leading to the stimulation of the coagulation/fibrinolytic system, which affects vascular permeability and nitric oxide production by endothelial cells [26]. This result suggests a possible peripheral molecular signature associated with immune function in early-onset schizophrenia patients. Moreover, according to the results of a meta-analysis focused on schizophrenia, absolute blood counts revealed a significant increase in total lymphocytes, CD3, and CD4, as well as in the CD4/CD8 ratio. CD3% was decreased in drug-native first-episode psychosis. Increased levels in CD4% and CD56% were observed in acutely relapsed subjects. These findings could be relevant in targeting a blood lymphocyte dysregulation signature for the initiation of antipsychotic medications [27]. 

C-Reactive Protein (CRP) is one of the main markers of inflammation. CRP modulation is a major marker of therapeutic response for anti-inflammatory treatment [19,28]. CRP is synthesized in the liver and released by macrophages and adipocytes in response to the stimulation of the expression of IL-1β, IL-6, and TNF-α [19]. Symptoms of schizophrenia and cognitive dysfunction are strongly associated with an increase in CRP [29,30]. Moreover, resistance to the treatment of schizophrenia is correlated with high levels of CRP [31]. 

The oxidative stress observed in schizophrenia is associated with the secretion of pro-inflammatory cytokines, leading to a chronic neuroinflammatory process and genetic damage that in turn promotes chronic inflammation, surrounding oxidative stress impacting cells, and immune impairment [1]. A vulnerability–stress–inflammation interaction model operates in schizophrenia. This model of schizophrenia includes the contribution of oxidative stress as a cause of increased genetic vulnerability in this pathogenesis. Oxidative stress can increase the secretion of pro-inflammatory cytokines, leading to a lasting pro-inflammatory state. Thus, the vulnerability–stress–inflammation relationship observed in schizophrenia may suggest that inflammatory response in the mother can induce genetic damages during pregnancy. This damage leads to deleterious effects on the neurological development of the fetus and can increase the risk of developing schizophrenia. Nevertheless, this hypothesis remains very uncertain to this day.

The association between oxidative stress and chronic inflammation increases the release of pro-inflammatory cytokines, the stimulation of astrocyte activity, dopaminergic and glutamatergic pathway dysregulation, and finally the onset of schizophrenia symptoms. The oxidative stress seen in schizophrenia interacts with inflammation [32], suggesting that the symptoms of schizophrenia are associated with specific changes in dopaminergic, serotonergic, noradrenergic, and glutamatergic neurotransmission. The dysfunction of the immune system can hinder the neurotransmission of dopamine and glutamate. The immune system can stimulate indoleamine 2,3-dioxygenase, an enzyme involved in the tryptophan/kynurenine mechanism [15]. In the brain, kynurenic acid acts as a natural antagonist of N-methyl-D-aspartate (NMDA). In the CSF of schizophrenic patients, kynurenic acid levels are increased [33]. Pro-inflammatory cytokines stimulate the concentration of kynurenic acid and lead to the production of antibodies to the NMDA receptor. This process supports the idea that the involvement of the glutamatergic pathway and NMDA receptor is major in schizophrenic patients [15,32].

## 3. The WNT/β-Catenin Pathway

The name WNT (Wingless-related integration site) is derived from Wingless drosophila melanogaster and its mouse homolog Int. The WNT/β-catenin pathway is involved in many regulatory pathways, such as embryogenesis, cell proliferation, migration and polarity, apoptosis, and organogenesis [34]. However, in several pathological conditions, the WNT/β-catenin pathway is disrupted, such as in inflammatory, metabolic, and neurological disorders; tissue fibrosis; and cancers [13,35,36].

The WNT proteins are members of the family of glycoproteins produced by modified lipids [37]. WNT ligands are produced by neurons and immune cells in the CNS [38]. The regulation of the WNT/β-catenin pathway leads to the involvement of embryonic development, cell fate, epithelial-mesenchymal transition (EMT), and metabolism. The formation of the complex composed by the transcriptional coactivator β-catenin and its transcriptional factor T Cell Factor/Lymphoid Enhancer Factor (TCF/LEF) is considered as a major step of the WNT/β-catenin pathway. The accumulation of β-catenin in the cytosol is modulated by the formation of the destruction complex composed of AXIN, glycogen synthase kinase-3 (GSK-3β), tumor suppressor adenomatous polyposis (APC), protein phosphatase 2A (PP2A), and casein kinase 1α CK1α. In the absence of WNT ligands, the destruction complex hyper-phosphorylates the cytosolic β-catenin and promotes its proteasomal degradation. In contrast, if they are present, WNT ligands complex with Frizzled (FZL) and low-density lipoprotein (LDL) receptor-bound protein 5/6 (LRP 5/6) to decrease the action of the β-catenin destruction complex. Thus, β-catenin transfers to the nucleus to bind to the TCF/LEF. This mechanism activates the different WNT target genes [39,40,41]. GSK-3β is a main inhibitor of the WNT/β-catenin pathway [42,43,44,45,46,47]. It is involved in the modulation of many pathophysiological pathways, such as cell membrane signaling, cell polarity, and inflammation [48,49,50].

### 3.1. WNT/β-Catenin Pathway and Schizophrenia

As mentioned previously, WNT/β-catenin pathway dysregulation leads to many deleterious effects in neuronal development, contributing to the pathogenesis of neurodevelopmental diseases, such as schizophrenia [51,52]. More specifically, a recent study indicated that the WNT/β-catenin pathway plays a major role in modulating dopaminergic (DA) activities [53]. The inhibition of GSK-3β activity and increased cytosolic accumulation of β-catenin promote the transformation of neuronal precursors into dopaminergic neurons, demonstrating that the WNT/β-catenin pathway can regulate neural fate in DA activity [54]. Furthermore, several authors have observed that the levels of β-catenin, APC, and the activity of GSK-3β are deregulated in the hippocampus of schizophrenic mice models [55,56,57]. Moreover, the WNT and FZD ligands are damaged in schizophrenia [58,59,60]. A genome-wide single-nucleotide polymorphism study showed that the deregulation of the membrane expression of FZD was associated with the onset of schizophrenia [61]. However, while FZD3 has been proposed as a possible marker of schizophrenic susceptibility [62,63], other studies have failed to support this hypothesis [64]. Nevertheless, these results may suggest that the disruption of the WNT/β-catenin pathway plays a major role in schizophrenia and may be a targeted pathway for its treatment.

### 3.2. Interplay between WNT/β-Catenin Pathway and Inflammatory Markers

The activation of the nuclear factor-kappa B (NF-κB) pathway and stimulation of the expression of cytokines and prostaglandins are responsible, in part, for neuroinflammation [65,66]. Numerous studies have shown the close relationship between the NF-κB pathway and the WNT/β-catenin pathway. This relationship operates through a negative interaction between the WNT/β-catenin and the NF-κB pathways. The WNT/β-catenin pathway decreases the activity of the NF-κB pathway [67,68,69], which controls several diverse targets, such as cytokines, chemokines, growth factors, immune receptors, transcription factors, and apoptosis repressors [70]. The NF-κB pathway is a key component of both acute and chronic inflammation. This pathway is composed of cytosolic-related transcription factors of two proteins (homodimers or heterodimers) of the Rel family of proteins, which are retained in the cytosol by an inhibitory protein called I-κB. The phosphorylation of I-κB causes the degradation of the inhibitor, leading to the release of NF-κB for translocation into the nucleus to activate its target genes. The activation of β-catenin signaling is associated with the inhibition of NF-κB pathway activity via NF-κB DNA binding arrest [70]. Other studies have shown that the inhibition of the GSK-3β activity leads to reduced activity of the NF-κB pathway [71]. GSK-3β activates the NF-κB pathway but decreases the level of β-catenin at the cytosolic level [72,73]. Thus, the activation of β-catenin, either directly or via the inhibition of GSK-3β, is associated with the reduction in NF-κB pathway activity and results in a decrease in target genes, including IL-6, IL-8, and TNF-α. This negative relationship may show a possible anti-inflammatory role of the WNT/β-catenin pathway [68,74].

To this effect, β-catenin signaling can complex with RelA and p50 to stop the NF-κB DNA binding and its transactivation activity. However, the protein–protein relationship between β-catenin and the NF-κB pathway is indirect. These two pathways do not directly bind to each other, as stimulated β-catenin decreases the expression of the NF-κB target gene named FAS [75]. Similarly, other studies focused on colorectal cancer cells have shown that stimulated β-catenin inhibits the NF-κB pathway through the activation of the phosphatidylinositide 3-kinase (PI3K) pathway [76]. This relationship is also observed in non-tumor cells, such as chondrocytes, fibroblasts, epithelial cells, osteoblasts, and hepatocytes [71]. 

### 3.3. Interplay between WNT/β-Catenin Pathway and Neuroinflammation in Schizophrenia

The disruption of the WNT/β-catenin pathway is observed in several diseases associated with chronic neuroinflammation, such as neurodegenerative diseases, but also in psychotic disorders, such as obsessive-compulsive disorder, depression, and autism spectrum disorder [8,11,77,78,79,80]. This deregulation is mainly observed in the cells of the CNS, such as macrophages/microglia, astrocytes, and oligodendrocytes. This suggests that the WNT/β-catenin pathway could be targeted to repair brain damage induced by neuroinflammation.

The inflammatory process stimulates peripheral immunoinflammatory pathways, such as IL-6, high-mobility box protein 1 (HMGB1), Dickkopf 1 (DKK1), and CCL11 (eotaxin) [81,82]. Thus, an increase in the expression of IL-6 leads to the overstimulation of both HMGB1 and DKK1 in schizophrenia [82]. Only when the WNT/β-catenin pathway is decreased can the neuroinflammatory process operate [81,83]. In parallel with the overstimulation of IL-6 and the decrease in IL-10, the disruption of the WNT/β-catenin pathway leads to the initiation of the neurotoxicity process in schizophrenic patients [82,84].

## 4. Interplay between Neuroinflammation and Oxidative Stress in Schizophrenia

**Figure 1:**
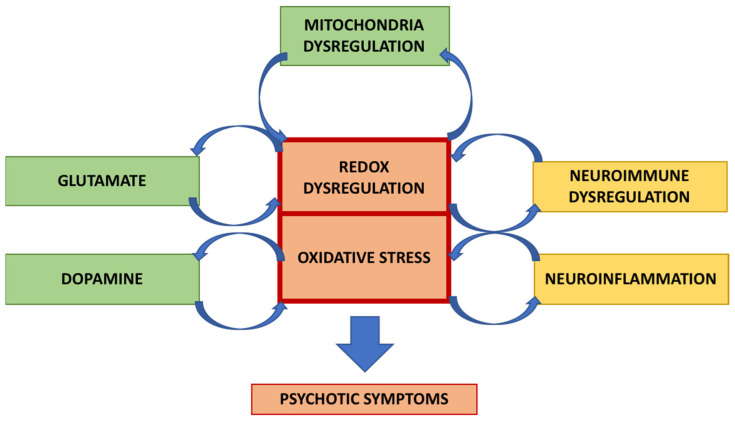
Schematic representation of the mechanism for the interaction between neuroinflammation and oxidative stress in schizophrenia. A vicious circle can occur in which these processes stimulate each other, leading to psychotic symptoms.

Previous studies have shown that a vicious and positive interplay operates between oxidative stress and neuroinflammation (Figure 1). 

Indeed, the activation of the NF-κB pathway leads to the production of prostaglandins and the development of oxidative stress [85]. Similarly, in a direct feedback, oxidative stress can activate the NF-κB pathway. Several inflammatory proteins, such as cyclooxygenase 2 (COX-2) and matrix metalloproteinase-9 (MMP-9), are stimulated by pro-inflammatory cytokines through reactive oxygen species (ROS)-dependent signaling in astrocytes [86].

A strong relationship between oxidative stress and the immune system has been shown to be involved in schizophrenia [1]. Glutathione (GSH), an antioxidant that is essential for the myelination and maturation of white matter, is stimulated by the amino acid precursor N-acetyl cysteine (NAC). NAC has many properties: antioxidant, anti-inflammatory, and the control of NMDA synaptic receptors. NAC supplementation improves positive symptoms and neurocognition in schizophrenia patients with high peripheral oxidative stress [87]. Omega-3 polyunsaturated fatty acids also exhibit antioxidant capacity and anti-inflammatory effects [88].

Pro-inflammatory cytokines are associated with the schizophrenic process via the disruption of major neurotransmitter systems [89]. Pro-inflammatory cytokines are associated with increased concentrations of kynurenic acid, a natural antagonist of NMDA receptors. Kynurenic acid is responsible for the inhibition of the NMDA receptor. The disruption in glutamatergic transmission leads to symptoms of schizophrenia [90]. Thus, neuroinflammation and oxidative stress are mainly interdependent. Indeed, tissue damage caused by oxidative stress could be directly responsible for neuroinflammation and immune response [91]. Macrophages and microglia use ROS to destroy pathogens [92]. This observation suggests that oxidative stress may be both the cause and the result of neuroinflammation [93]. Oxidative stress activates the NF-κB pathway, which leads to an increased production of more free radicals [94]. However, the immune system is primarily a source of oxidative stress due to the activation of microglia using NADPH oxidase to produce reactive superoxide to destroy pathogens [95].

## 5. Oxidative Stress in Schizophrenia

Oxidative stress is a well-known process in schizophrenia [84,96]. Although this may not be the leading cause of schizophrenia, new results have suggested that oxidative stress may participate in the declining course of schizophrenia [97].

A large number of studies have evaluated peripheral biomarkers of oxidative stress, including antioxidant levels. Recent studies have observed that oxidative stress biomarkers, such as GSH, are at low levels in the plasma of schizophrenic patients [98,99]. In contrast, high levels of ROS have been found in schizophrenic patients [100]. The increase in ROS is strongly correlated with a decrease in superoxide dismutase (SOD) and glutathione peroxidase (GPx) levels [101]. The phases of the disorder, acute or stable, modulate the redox regulation [102]. Post-mortem studies have shown that there is a reduction in GSH levels in the prefrontal cortex and caudate of schizophrenic patients [103,104], showing that oxidative stress is expressed via the abnormal expression of GSH in the anterior cingulate cortex (ACC) [105]. Decreased levels of GSH in the blood are associated with the onset of symptoms of psychosis and alterations in brain volume in schizophrenia [106]. Moreover, as previously discussed, the administration of NAC in combination with antipsychotic therapies could soothe the symptoms of schizophrenia [107].

Furthermore, redox dysfunction leading to oxidative stress in schizophrenia [102,108] is characterized by the low expression of polyunsaturated fatty acids (PUFAs) in red blood cells during the acute phase of schizophrenia [108]. This process is also observed during the stable phase of schizophrenia, with a high expression of 2-amino butyrate in the low PUFA group showing persistent redox dysregulation [102]. Thus, the process of oxidative stress leads to neuronal excitability through the disruption of mitochondrial functions in schizophrenia [109]. To this end, the deficiency in antioxidant defense in schizophrenic patients could be associated with the appearance of aggravated symptoms [110]. 

## 6. The Glutamate Hypothesis of Oxidative Stress in Schizophrenia

Rapid excitatory neurotransmission is modulated in the CNS by glutamate. In neurons, glutamate is stored in synaptic vesicles, from which it is released. The release of glutamate leads to a sharp increase in its concentration in the synaptic cleft, which connects the ionotropic glutamate receptors. Glutamate is then removed from the synaptic cleft to be transported to the astrocytes by glutamate transporters (such as GLT-1 or excitatory amino acid transporters 1 and 2: EAATs 1 and 2) to prevent the upregulation of glutamate receptors [111]. Astrocytes remove more than 90% of excess glutamate by EAATs and play a major role in the glutamate/glutamine cycle. After glutamate absorption, glutamine synthetase (GS) catalyzes the ATP-dependent reaction of glutamate and ammonia into glutamine. Glutamine is released and, in turn, is absorbed by neurons to be converted into glutamate by glutaminase. 

Schizophrenic patients may present with low levels of glutamate in the CSF [112]. This hypothesis of the role of glutamate in schizophrenia could indicate that the negative symptoms observed are partly caused by a dysfunctional glutamatergic pathway, and thus are directly modulated by NMDA receptors on GABAergic interneurons [113].

**Figure 2:**
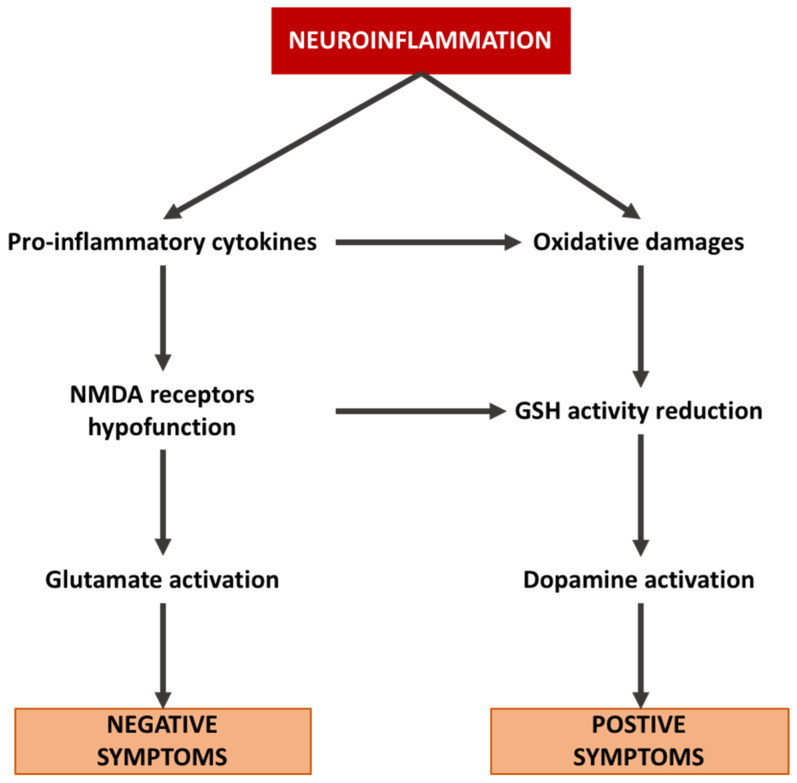
Interaction between neuroinflammation, glutamate pathway, dopamine pathway, and subsequent stimulation of positive and negative symptoms in schizophrenia.

GSH shows a protective role in protecting cells from ROS damage generated by dopamine (DA) metabolism. The observed deficit in GSH results in membrane peroxidation and lesions forming around dopaminergic terminals, leading to a loss of connectivity [114]. Furthermore, DA neurons are controlled by glutamatergic projections to the midbrain DA nuclei [115]. In schizophrenia, DA function is altered by glutamate function [116], while NMDA blockers, such as ketamine, can increase the activation of the DA system [117]. Thus, the NMDA hypofunction observed in schizophrenia may enhance the DA system to be more sensitive to the effects of oxidative stress [118] (Figure 2). 

Thus, to corroborate this hypothesis, it turns out that NMDA receptor antagonists, such as ketamine, can lead to psychosis in healthy participants [119]. This psychosis caused by said antagonists reveals symptoms close to those of schizophrenia [120]. Increased GSH levels stimulate the response of NMDA receptors, while the inhibition of GSH leads to decreased NMDA receptor activity [121]. The inhibition of the activity of these receptors is associated with an increase in the production of free radicals and therefore the enhancement of oxidative damage [122]. For this purpose, it should be noted that the synaptic activity of NMDA receptors is mainly related to the production of GSH [123].

## 7. Interplay between Glutamate and the WNT/β-Catenin Pathway

Physiologically, within astrocytes, β-catenin stimulates the gene expression of EAAT2 and GS [124]. This stimulation is responsible for the reuptake of glutamate from the synaptic cleft by EAAT2 to the astrocytes. Then, GS metabolizes glutamate within these astrocytes. 

The β-catenin pathway directly controls the expression of EAAT2, GLT-1, and GS [124,125,126,127]. Thus, the complete inhibition of the β-catenin signaling leads to the cessation of glutamate neurotransmission [125]. The expression of EAAT2 and GS is also modulated by the direct interaction between the β-catenin and its target TCF/LEF [124]. It should be noted that numerous studies have shown the possible role that the NF-κΒ pathway can play in modulating the expression of EAAT2 [126].

A vicious circle can occur between a decrease in the expression of β-catenin, a decrease in the activity of EAAT2 and GS, neuroinflammation, and the excitotoxicity of glutamate [124,127]. 

## 8. PPAR α Agonists: Possible Therapeutic Targets in Schizophrenia

Few effective treatments exist for schizophrenia, so it is essential to find new therapeutic pathways. One possible interesting pathway is the use of peroxisome proliferator-activated receptor (PPAR) agonists through their multiple applications in neuroinflammation, oxidative stress, and their interaction with the WNT/β-catenin pathway.

PPARs are ligand-activated transcription factors that bind PPRE (PPAR response elements). PPARs are subdivided into three isoforms: PPARα, PPARγ, and PPAR β/δ [128]. In the nucleus, PPARs form a heterodimer with the retinoid X receptor (RXR) [129]. They are composed of a ligand binding domain that interacts with a DNA binding domain to modulate it [130]. PPARs are involved in several pathophysiological mechanisms, including cell differentiation, protein metabolism, lipid metabolism [131,132], adipocyte differentiation, insulin sensitivity, and inflammation [133,134].

Recent studies have shown that PPARα may modulate the neuroinflammatory mechanisms observed in psychiatric diseases, such as depression [135] and schizophrenia [136]. In schizophrenia, the expression of PPARγ could be increased while PPARα is downregulated, suggesting a metabolic-inflammatory imbalance in its pathogenesis [137]. Note that PPARγ is well known to be expressed inversely to PPARα [138]. The prenatal administration of pioglitazone in a maternal immune rat model, a PPARγ agonist, could attenuate the schizophrenic behavior observed in the male [139]. However, rosiglitazone, another PPARγ agonist, has no significant beneficial effect on the cognitive performance of schizophrenic patients [140]. These contrasting results could be explained by the differences in posology, treatment, and populations. However, stimulating PPARγ would not be the expected therapeutic solution in schizophrenia because of its observed stimulation in schizophrenia. 

The decrease in PPARα expression is associated with the development of schizophrenia [141]. It has been shown in numerous studies that the stimulation of PPARα is associated with a decrease in NF-κB pathway activity [142], the downregulation of TNF-α [143], and a decrease in the production of pro-inflammatory cytokines and interferons [144]. Thus, PPARα agonists could play an interesting role in decreasing the neuroinflammation observed in schizophrenia. For this purpose, the stimulation of PPARα increases the brain synthesis of oleoylethanolamide (OEA) and palmitoylethanolamide (PEA) [145]. Both these molecules have anti-inflammatory properties [139]. In addition, one study showed that the decrease in PPARα mRNA levels is concomitant with the increased expression of IL-6 and TNFα [146]. A functional polymorphism L162V in the PPARα gene was observed in a subgroup of schizophrenic patients [147].

**Figure 3:**
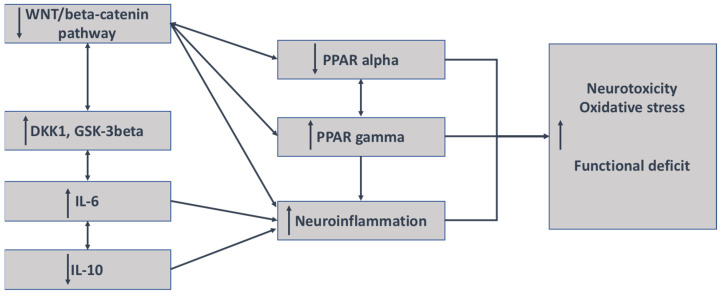
Mechanisms of interaction between neuroinflammation and the WNT/β-catenin pathway in schizophrenia. The decrease in the WNT/β-catenin pathway is associated with the increase in both DKK1 and GSK-3 β and their inhibitors; the stimulation of IL-6 and IL-8 expression; and a decrease in the expression of IL-10, an antagonist marker of inflammation. The decrease in WNT/β-catenin leads to the upregulation of PPARγ but the downregulation of PPAR α. An increase in the expression of PPARγ leads to the involvement of neuroinflammation.

Numerous studies have shown that the WNT/β-catenin pathway and PPARγ act in an opposite manner, including psychiatric disorders, neurodegenerative diseases, and fibrosis processes [80,148,149,150,151,152,153] (Figure 3).

In many diseases, the expression of PPARγ is downregulated by the overexpression of the WNT/β-catenin pathway [154,155,156]. PPARγ is considered a β-catenin target, and when targeted becomes inhibited [157,158]. The WNT/β-catenin pathway and PPARγ interact through a TCF/LEF domain of β-catenin and a catenin binding domain in PPARγ [159,160]. The β-catenin pathway stimulates the PI3K/Akt pathway, leading to a decreased PPARγ expression in 2T2-L1 adipocytes and preadipocytes [161,162]. Recent studies have shown a positive interaction between the WNT/β-catenin pathway and PPARα, elucidating the possible action of this crosstalk to reduce PPARγ expression.

## 9. Conclusions

A growing body of evidence has shown the important role of neuroinflammation in the pathogenesis of schizophrenia. In addition, the relationship between neuroinflammation and oxidative stress is well documented in this disorder. The WNT/β-catenin pathway is essential for several cellular events that take place in development, homeostasis, and regeneration. However, there is limited knowledge regarding its dysregulation in schizophrenia. Therefore, further studies are still needed in order to clarify the role of the WNT/β-catenin pathway in schizophrenia. Understanding the molecular mechanism of the WNT/β-catenin pathway will contribute to the development of novel therapeutic strategies for schizophrenia. Recently, new evidence has been provided on the protective effects of activation of PPARα expression. In schizophrenia, the WNT/β-catenin pathway and the expression of PPARα act in a positive interplay, which in turn leads to this complex negatively interacting with the expression of PPARγ. This triptych could be a new therapeutic approach to counteract neuroinflammation and oxidative stress in schizophrenia. 

## Data Availability

Not applicable.
